# Superior Vena Cava Syndrome in a Patient with Polycytemia Vera: Diagnosis and Treatment

**DOI:** 10.4061/2010/791648

**Published:** 2010-06-03

**Authors:** Salvatore Lentini, Mario Barone, Filippo Benedetto, Francesco Spinelli

**Affiliations:** Cardio vascular and Thoracic Department, Policlinico G. Martino, University of Messina, Viale Gazzi, 98100 Messina, Italy

## Abstract

Polycythemia vera is a myeloproliferative disorder characterized by thrombotic complications both in the arterial and venous systems. We report the case of a 55-year-old patient affected by polycythemia vera, presenting with acute superior vena cava syndrome due to thrombosis of the upper part of the superior vena cava. Diagnosis was done clinically and by computed tomography scan and showed an unusual finding: an air bubble trapped in the brachiocephalic venous trunk. The patient underwent emergency surgery. Diagnosis and treatment of the case are discussed.

## 1. Introduction

Polycythemia vera is a chronic myeloproliferative disorder characterized by complicated thrombotic and hemorrhagic events [1]. The diagnosis and treatment of this condition remain controversial [2].

## 2. Case Report

A 55-year-old male patient was referred from another hospital, acutely unstable and with signs of superior vena cava (SVC) syndrome. He had a history of polycythemia vera and was on anticoagulation therapy with warfarin and cytoreduction therapy with hydroxyurea.

Physical examination showed that body temperature was 36.4°C, pulse was 115/min, respiration was 22/min, blood pressure was 80/55 mm Hg, and arterial oxygen saturation was 87%. He had jugular venous distension, head and neck oedema, basal pulmonary rales, palpable splenomegaly, and no heart murmurs. He did not have any sign of bleeding tendency. Laboratory examinations revealed that hemoglobin was 16.16 g/dL, red blood cell count was 5.97 M/*μ*L, neutrophilia was 8.54 K/*μ*L, platelet count was 85 K/*μ*L, hematocrit was 46.30%, and lactate dehydrogenase (LDH) was 486 U/L. His INR was 2.7. The patient had a previously implanted cardiac pacing device, with leads passing through the brachiocepahalic vein to reach the right atrium and right ventricle.

**Figure 1:**
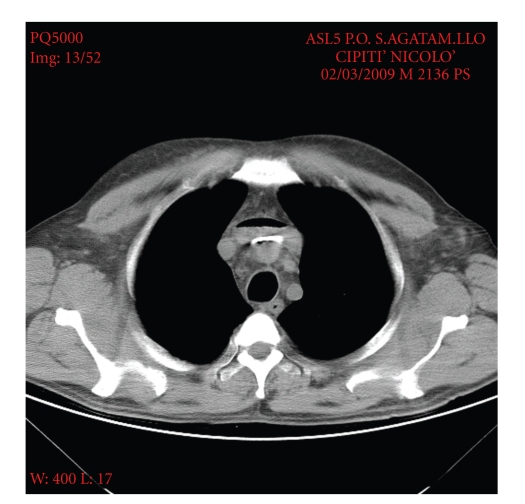
CT scan showing air bubbles trapped at the level of the venous brachiocephalic trunk.

A CT scan showed air bubbles trapped at the level of the venous brachiocephalic trunk (Figure 1). The azygos and hemiazygos veins appeared enlarged. No masses compressing the SVC were detected. Echocardiogarphy showed no intracardiac masses.

Due to a rapidly deteriorating condition, no other investigations were attempted. The patient was transferred to the operating room for emergency surgery with a diagnosis of acute SVC obstruction in a patient with polycythemia vera.

**Figure 2:**
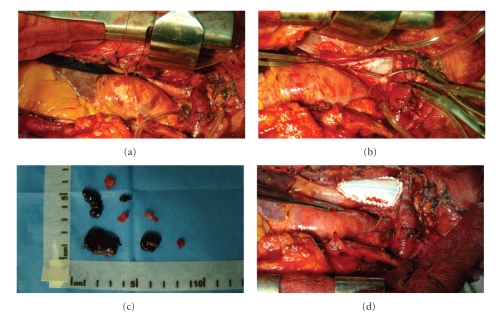
Surical findings. (a) The venous brachiocephalic trunk appeared enlarged. (b) The SVC and brachiocephalic vein were clamped. Their junction is opened, and thrombectomy performed. (c) A collection of samples of thrombus. (d) Venous repair using an enlargement patch of GoreTex.

At surgery (Figure 2), through median sternotomy, the venous brachiocephalic trunk appeared enlarged. The SVC and brachiocephalic vein were clamped. A large thrombus was found at the junction of the SVC with the brachiocephalic trunk, where the pacing leads passed through. SVC flow was maintained through the azygos vein. Thrombectomy and venous repair using an enlargement patch of GoreTex (W.L. Gore & Associates, Newark, DE) resolved the acute event. 

Postoperative course was uneventful. The patient was discharged on postoperative day 8 and remains clinically well at 6-month follow-up.

## 3. Discussion

Polycythemia vera is a myeloproliferative disorder. Patients with such pathology are at risk for thrombotic complications in both the arterial and venous systems [1]. Intracardiac thrombosis has been reported as well [2, 3]. An increase in circulating neutrophil or platelet aggregates has been noted in such patients [4]. The reported patient was at high risk for venous thrombosis, due to both the polycythemia vera and the presence of pacing leads on the right venous side of the heart. 

The diagnosis in this patient was done mainly clinically but also with the use of the CT scan. On the scan a particular finding was present: air bubbles trapped in the brachiocephalic vein, probably arising from intravenous injection before performing the CT scan. The bubbles remained trapped in the brachiocephalic vein due to the obstruction of the upper part of the SVC. The lower part of the SVC was however patent, with flow from the azygos vein.

Upper body oedema of rapid onset and air bubbles trapped in the brachiocephalic trunk, as seen on the CT-scan, should be regarded as suspicious signs for acute vena cava syndrome in patients at risk for venous thrombosis (myeloproliferative disorders), especially in the presence of concomitant venous catheters, and should strongly encourage rapid treatment.

Successful treatment with streptokinase has also been reported. However, in the present case, surgery was mandatoary due to the rapidly deteriorating condition [5].

Therapy for thrombosis prevention in such patients is still debated. Several platelet antiaggregation medications have been proposed, but with no consensus yet formed [6].

## References

[1] Landolfi R, Cipriani MC, Novarese L (2006). Thrombosis and bleeding in polycythemia vera and essential thrombocythemia: pathogenetic mechanisms and prevention. *Best Practice and Research: Clinical Haematology*.

[2] Venegoni P, Cyprus G (1994). Polycythemia and the heart: a review. *Texas Heart Institute Journal*.

[3] Yuan S-M, Shinfeld A, Raanani E (2009). Massive intraventricular thrombus in polycythemia vera. *Journal of Cardiac Surgery*.

[4] Falanga A, Marchetti M, Barbui T, Smith CW (2005). Pathogenesis of thrombosis in essential thrombocythemia and polycythemia vera: the role of neutrophils. *Seminars in Hematology*.

[5] Al-Saif S, Bhat RP, Hijazi A, Al-Faraj A, Quadri I (1996). Left ventricular and aortic valve thrombosis caused by polycythemia rubra vera successfully treated with streptokinase. *American Heart Journal*.

[6] Landolfi R, Di Gennaro L (2008). Prevention of thrombosis in polycythemia vera and essential thrombocythemia. *Haematologica*.

